# Recent Applications of Amphiphilic Copolymers in Drug Release Systems for Skin Treatment

**DOI:** 10.3390/pharmaceutics16091203

**Published:** 2024-09-13

**Authors:** Yudy Vanessa Cardona, Lizeth Geraldine Muñoz, Daniela Gutierrez Cardozo, Andrés Felipe Chamorro

**Affiliations:** Research Group of Electrochemistry and Environment (GIEMA), Faculty of Basic Sciences, Universidad Santiago de Cali, Cali 760035, Colombia

**Keywords:** skin diseases, cancer, amphiphilic copolymers, nanomaterials, polymers, drug-controlled release

## Abstract

Amphiphilic copolymers (ACs) are versatile systems with self-assembling and aggregating properties, enabling the formation of nanomaterials (NMs) such as micelles, vesicles, nanocapsules, and nanogels. These materials have been extensively explored for the delivery of various drugs and active compounds, enhancing the solubility and permeation of poorly water-soluble drugs into skin tissue. This improvement facilitates the treatment of skin diseases, including chronic conditions like cancer, as well as infections caused by bacteria, fungi, and viruses. This review summarizes recent applications of ACs in skin treatment, with a particular focus on their use in anti-cancer drug therapy. It covers the synthesis, classification, and characterization of ACs using various experimental techniques. Additionally, it discusses recent research on different drug delivery pathways using ACs, including encapsulation efficiency, release behavior, characteristics, applications, and responses to various chemical and physical stimuli (both in vivo and in vitro). Furthermore, this review provides a comprehensive analysis of the effects of ACs NMs on several skin diseases, highlighting their potential as alternative treatments.

## 1. Introduction

The skin is the largest organ of the human body, serving as a protective barrier for other systems. However, it is highly susceptible to pathogens and environmental factors, increasing its vulnerability to diseases [1]. For instance, fungi, viruses, and bacteria can lead to various skin conditions and diseases such as cellulitis, impetigo, cutaneous mycosis, and dermatitis. Additionally, the skin can be affected by genetic alterations that result in chronic diseases like cancer, one of the most severe conditions caused by mutations in genetic material, leading to the uncontrolled reproduction of skin cells [2]. Basal cell carcinoma, squamous cell carcinoma, and melanoma are the most common types of skin cancer. According to the World Health Organization, in 2022, melanoma alone accounted for approximately 58,667 deaths worldwide, with Europe showing the highest mortality (26,180 deaths) and the highest incidence of cases (146,321) [3]. Common treatments for skin cancer include chemotherapy and surgery; however, these are often invasive and can negatively impact the patient. Drug application through the skin has emerged as a promising alternative for cancer treatment due to the skin’s large surface area (1.8–2.0 m^2^) [4], enabling topical, dermal, or transdermal applications. The latter two methods are particularly beneficial for targeting the skin layers, as they minimize the dosage required compared to oral or parenteral administration [5]. 

In the last 20 years, advancements in nanotechnology have led to the development of new methodologies aimed at improving drug effectiveness while reducing side effects [6]. The International Union of Pure and Applied Chemistry (IUPAC) defines NMs as particles of any shape with an equivalent diameter ranging from approximately 1 to 100 nm [7]. These NMs are considered effective in enhancing the permeation of drugs through the skin layers, thereby targeting cancer cells more efficiently [8]. Human skin is a multilayered structure composed of the epidermis, dermis, and hypodermis, with the epidermis itself consisting of multiple layers, including the stratum basale, stratum spinosum, stratum granulosum, stratum lucidum, and the stratum corneum (SC) [8]. The SC, a thick keratinized epithelium, poses a significant barrier to the transdermal delivery of both hydrophobic and hydrophilic drugs. This barrier function is crucial for protecting the body against harsh environmental conditions [8,9]. 

NMs can be metallic, polymeric, inorganic, and/or hybrid, with their composition significantly influencing their interaction with the skin. For instance, gold (Au) NMs have been shown to penetrate the skin of adult rats, particularly across the SC. The permeation of these particles depends on their size, with Au NMs of 22 nm exhibiting greater penetration compared to those measuring 105 nm and 186 nm. Additionally, after four days of contact, the presence of these NMs was detected in the bloodstream, indicating that they had traversed both the dermis and hypodermis [9]. The dermis is a skin layer composed of pilosebaceous units, blood vessels, hair follicles, and sweat glands [10], while the hypodermis, a subcutaneous tissue made up of adipose tissue, serves as a reservoir of energy due to its high fat content [5]. Other studies have demonstrated that the charge and shape of NMs also affect skin permeation. For example, Lee et al. (2013) found that anionic Au NMs showed higher skin penetration than cationic Au NMs [11]. Similarly, the shape of NMs can impact their ability to permeate the skin; Friedman et al. (2021) showed that Au nanostars had higher penetration in human skin than spherical particles [12]. On the other hand, the chemical composition of NMs is crucial for their application in drug delivery. Unlike Au NMs, titanium dioxide (TiO_2_) and zinc oxide (ZnO) nanoparticles (NPs) have been reported to not penetrate the deeper layers of human and porcine skin (epidermis and dermis), remaining localized in the SC [13]. However, Cross et al. (2007) reported limited penetration by small-sized ZnO NPs (20–30 nm), where only 0.03% of the total applied dose was internalized [14]. 

**Figure 1:**
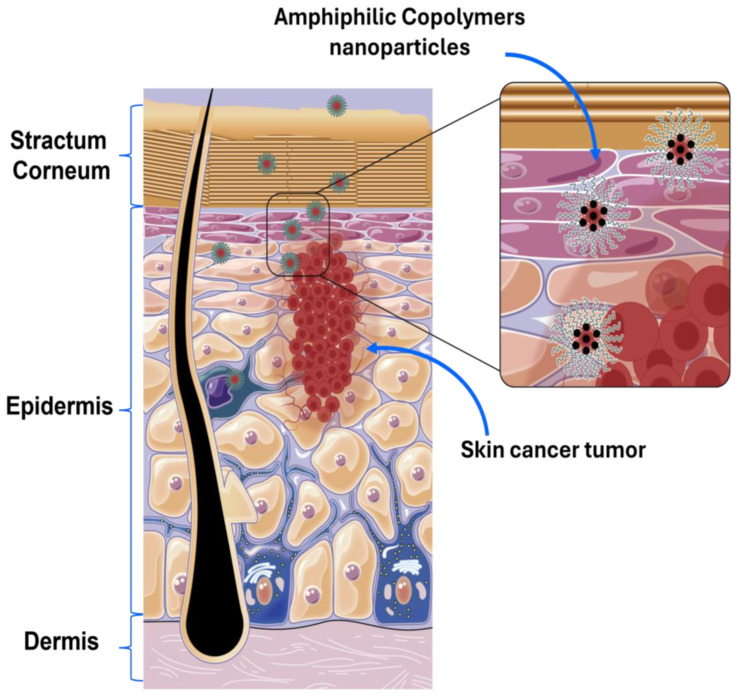
Schematic representation of ACs NPs through the ski multilayered structure with cancer tumor. The polymeric NPs can penetrate the skin layers and release active compounds in the cancer cells, improving the treatment.

Polymeric NMs, have been the focus of extensive research, particularly ACs, which consist of both hydrophilic and hydrophobic polymer chains. These ACs exhibit self-assembling and aggregating properties in selective solvents, forming NMs such as nanocapsules, nanoemulsions, micelles, nanogels, and more [15]. ACs are especially attractive for various pharmacological applications, largely due to their amphiphilic nature, which allows them to encapsulate a greater quantity of hydrophobic compounds in an aqueous medium compared to metallic NPs. This capability makes AC NMs particularly suitable for hydrophobic drug delivery in cancer and skin treatments. AC NMs can penetrate the skin through several pathways, including via dermal structures such as hair follicles, intracellularly through corneocytes, and intercellularly around corneocytes (Figure 1) [10]. The route of penetration depends on factors such as the NMs’ composition, size, charge, and morphology. Moreover, AC nanostructures are sensitive to external stimuli (e.g., temperature, pH, and medium composition), which allows for controlled drug release. In this context, this review explores the application of AC NMs in drug delivery for the treatment of skin diseases, particularly skin cancer. It covers the classification and characterization of ACs, in vitro anticancer drug encapsulation and release assays, their advantages and disadvantages, and in vitro and in vivo results in biological models.

## 2. Amphiphilic Copolymers

**Figure 2:**
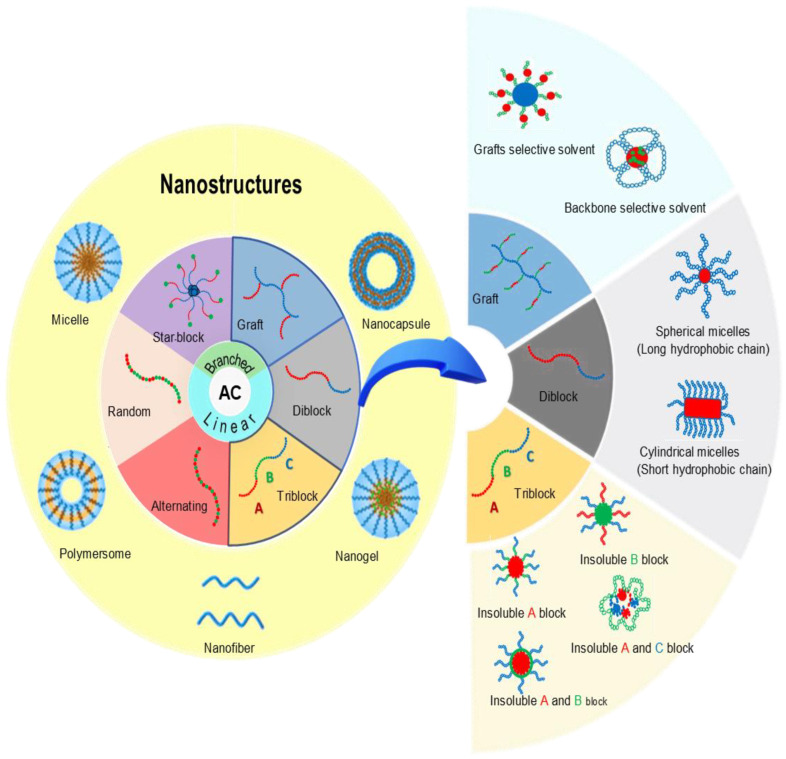
Branched and linear AC classification based on topology and composition (colorful pie chart). Nanostructures formed by different ACs are shown in yellow circumference. Basic micellar morphologies depending on selective solvents, for triblock or graft copolymers, and length of the hydrophilic segment (for deblock copolymers) are shown in the half colorful pie chart. Adapted from [17,20].

The ACs are characterized by their surfactant properties, enabling them to modify the surface tension of solutions in which they are dissolved. This property allows ACs to self-assemble into stable nanostructures such as micelles, vesicles, nanocapsules, and nanoemulsions under specific environmental conditions (Figure 2) [16,17,18,19,20]. These macromolecules consist of both hydrophobic and hydrophilic segments within their chemical structure. Different types of ACs can be synthesized based on the chemical nature and relative distribution of these hydrophilic and hydrophobic segments (Figure 2): (i) block ACs: These are created by the polymerization of two (diblock), three (triblock), or more segments with opposing affinities for aqueous solvents—one hydrophilic (neutral or ionizable) and the other hydrophobic [21]; (ii) alternating ACs: formed by an alternating distribution of a hydrophilic segment, which can be neutral or ionizable, and a hydrophobic segment, which can be aliphatic or aromatic [22]; (iii) statistical or random ACs: these systems have monomers that do not follow an ordered pattern in their sequence [22]; (iv) star-block ACs: these have a central structure with several covalently linked copolymeric arms, which may be identical or different [23]; and (v) graft or branched ACs: these consist of several linear polymer chains connected in a random, alternating, or block sequence to a linear polymeric backbone of a different chemical nature [24]. 

To obtain various forms of ACs and specifically control their length and composition—essential for achieving desired properties in the final copolymer—several techniques have been developed. For block copolymers, techniques such as anionic polymerization, reversible addition-fragmentation chain transfer (RAFT), and ring-opening polymerization (ROP) are commonly used [25,26]. Alternating copolymers require more complex synthesis due to the need to precisely control the sequence of monomer addition. Common methods for synthesizing alternating copolymers include charge transfer polymerization (CTP), polymerization with specific catalysts that promote strict alternation of monomers and controlled radical polymerization [27]. For statistical copolymers, free radical polymerization and RAFT polymerization are the most commonly employed techniques [28,29]. In the case of star block copolymers, methods such as living polymerization, living anionic polymerization, coupling reactions to link preformed polymeric arms to a central core, and ROP are utilized [30].

The length of the hydrophilic and hydrophobic segments, as well as the chemical structure of ACs, are critical parameters that influence the final nanostructures and their properties [16]. Various studies have explored the relationship between copolymer composition, sequence distribution, backbone, and chain length with the size and structure of nanoaggregates [31,32,33,34]. Key findings include the following: (i) The size of nanoaggregates is determined by the composition of monomer segments [31]. (ii) The radius of spherical NPs inversely depends on the number of monomers in the material chains, although it is independent of the molecular weight of the chains [33]. (iii) For a given copolymer composition, NPs are larger when there are more hydrophobic comonomers [34]. (iv) The hydrophobic and hydrophilic nature of AC components allow them to be utilized in diverse applications. For example, some drug efflux systems in multidrug-resistant cells are inhibited by Pluronic family copolymers when they are hydrophobic, while hydrophilic components can enhance cell viability [32]. The versatility and self-assembly capabilities of ACs make them ideal for various applications. Consequently, numerous studies are focusing on designing structures with tailored characteristics for use in fields such as biomedical applications (particularly in drug encapsulation and release), materials science, and nanotechnology.

On the other hand, characterizing these ACs and their nanoaggregates is crucial for understanding their properties and behaviors. Common characterization techniques include nuclear magnetic resonance spectroscopy (NMR) used to determine chemical structure and composition, gel permeation chromatography (GPC) to know molecular weight distribution, transmission electron microscopy (TEM), scanning electron microscopy (SEM) and atomic force microscopy (AFM) to visualize the morphology of nanoaggregates, dynamic light scattering (DLS) to measure the size and distribution of aggregates, and infrared spectroscopy (IR) and Raman spectroscopy to identify functional groups and study molecular interactions. These techniques offer a comprehensive view of the structural, chemical, and physical characteristics of ACs and their nanostructures, facilitating their effective design and application in various fields, from nanotechnology to biomedicine [27,29,30]. Below are some recent studies that utilize ACs for biomedical purposes and their respective characterization.

Table 1 displays various types of ACs recently synthesized, along with their prospective application fields, such as biomedical uses and nanocarriers. It also describes their nanostructure properties and characterization as well as the advantages and disadvantages of each type of AC. Depending on their classification, the intra- and inter-polymer aggregation behaviors of these copolymers can vary in aqueous media. Several examples of ACs are shown below, categorized according to their classification.

### 2.1. Block ACs

Block copolymers tend to separate into microphases due to block incompatibility. In the case of diblock ACs, this can result in the formation of different morphologies, such as micelles, cylinders, or sheets, depending on the proportions of the blocks and the processing conditions [35]. ACs can form micelles that encapsulate hydrophobic drugs, such as cannabis-based analgesics like tetrahidrocannabinol (THC). This encapsulation protects the drugs until they reach their destination, allows for controlled release, improves bioavailability, and reduces side effects [36]. Researchers used fluorescence and proton nuclear magnetic resonance (^1^H NMR) spectroscopy to confirm the formation of polymer micelles with nanometer-scale sizes, uniform distribution, and colloidal stability. GPC studies indicated that the number-average molecular weight of the micelles was 7.5 kDa [36].

**Table 1:** Prospective applications, nanostructure properties, and characterization of AC.

Formulation	Application	Nanostructure Properties and Characterization	Ref.	Advantages and Disadvantages
Block Amphiphilic Copolymers				Advantages Can form various morphologies (e.g., micelles, cylinders, sheets) due to its microphase separation. Capable of encapsulating hydrophobic drugs, protecting them until delivery. Allow for controlled drug release, enhancing bioavailability. Reduce drug side effects. Disadvantages: Block incompatibility may complicate processing due to microphase separation. Requires precise control of block proportions and processing conditions.
mPEG and copolymer of caprolactone and carbonate containing fatty acid (La-ABC and Ol-ABC)	Encapsulate the Δ^9^-tetrahydrocannabinol-rich extract to analgesic activity.	Fluorescence and ^1^H NMR studies demonstrated that the micelles have nanometer-scale sizes, even distribution, and colloidal stability. GPC analysis indicated that the number-average molecular weight was 7.5 kDa.	[36]	
Pluronic-based polymersomes	Encapsulation of enzymes as well as regulation of substrates without the use of biopores.	Confocal microscopy showed that these Pluronic-based polymersomes have a uniform size, with an average diameter of 118 μm.	[37]	
PLLA and PCL reinforced with βTCP or hCAp and pCAp	Composite materials for bone surgery and orthopedics.	SEM images showed a hexagonal prismatic (hCAp) shape with a width-to-height ratio of 0.8–1 and a width of 4–8 μm. The plate-like crystallites (pCAp) exhibited uniform size and morphology. The B-type CAp identity was confirmed by X-ray diffraction studies. Thermogravimetric analysis (TGA) and FTIR data indicated higher incorporation of CO₃²⁻ ions in hCAp compared to pCAp.	[38]	
PBMA-b-PMAA-b-PBMA	Medical and sensory materials.	Hydrogels exhibited changes in microstructure from spherical to cylindrical to lamellar forms as the molar fraction of the B-block decreased. Small angle X-ray scattering (SAXS) measurements determined that the radius of the structural domains was approximately 75.6 nm, and the interdomain space was around 15.5 nm.	[39]	
Alternating Amphiphilic Copolymers				Advantages: Can form micelle and vesicle nanostructures in water, as well as reverse micelles in non-polar solvents. Capable of loading hydrophilic drugs. Regular alternation enhances compatibility and miscibility compared to random copolymers. Potential applications as emulsifiers and stabilizers. Disadvantages: Its synthesis is complex and requires specific conditions to achieve the desired structures.
P(MF-alt-VBP)	Hydrophilic drug loading abilities.	DLS and TEM analyses indicate the formation of micelle and vesicle nanostructures, consistent with the amphipathic nature of the side chains. The copolymers exhibited spherical morphologies, with average sizes of 82 ± 18, 108 ± 22, and 48 ± 12 nm.	[40]	
Styrene–maleic acid	Emulsifiers and stabilizers.	Fourier transform infrared (FTIR) spectroscopy and NMR analyses revealed interactions between lipids, and ACs. Size exclusion chromatography (SEC) and DLS analyses reported a nanodisc size of 10 nm.	[41]	
Star Block Amphiphilic Copolymers				
PCL-Fc and POEGMA with different degrees of polymerization initiated by b-cyclodextrin	Drug carriers.	^1^H NMR and SEC-MALLS analyses confirmed the synthesis of star copolymers. Micelle nanostructures with 12-arm star-shaped copolymers exhibited uniform size and higher stability compared to the 9- and 18-arm compositions, which showed larger sizes as determined by DLS analyses.	[42]	Advantages: Offers high functionality and chain density. Provides superior encapsulation ability compared to linear copolymers. Can enhance physical, chemical, and mechanical properties (e.g., viscosity, solubility, wear resistance). Has potential for encapsulating compounds in non-polar media. Disadvantages: Synthesis is complex due to advanced properties.
pOEGMA-Br and pOEGMA-b-pLMA-Br	Transdermal carrier capabilities.	Reverse micelles, with a hydrophilic interior and hydrophobic exterior and covalently linked structures, were analyzed. DLS analyses showed aggregate sizes ranging from 175 to 250 nm for the 1-arm copolymer, while TEM analysis revealed spherical structures with sizes between 700 and 800 nm. According to both TEM and DLS analyses, the aggregates observed for the 6- and 12-arm polymers were smaller.	[43]	
Statiscal Amphiphilic Copolymers				
PBAx-co-PNMAy	Design of materials with elasticity, self-crosslinking, and self-healing characteristics.	^1^H NMR analysis revealed copolymer system ratios ranging from 8:2 to 7:3. SEC analysis determined the number-average molecular weight to be between 11,100 and 15,000. FTIR analysis confirmed the self-cross-linking behavior of the copolymers.	[44]	Advantages: Can exhibit a wide range of mechanical properties from rigid to flexible. Capable of forming unique assemblies (e.g., vesicles, spheres, hydrogels). Mechanical properties can be easily adjusted by modifying monomer ratios. Disadvantages: Variability in composition may lead to unpredictable properties. Phase separation may not occur unless the components are highly dissimilar.
PDMAEMA and PDIPAEMA	Nanocarriers of pharmaceutical compounds.	SEC, ^1^H NMR, and FTIR analysis identified the chemical structure of copolymers with random compositions that form various aggregates.	[45]	
Graft Amphiphilic Copolymers				
PCL-graft-pHPMA	Encapsulation and delivery of hydrophobic drugs.	SEC, DLS, ^1^H NMR, and field flow fractionation (FFF) analyses showed the formation of micelles with a hydrodynamic diameter of approximately 25 nm. The critical micelle concentration (CMC) was found to be low.	[46]	Advantages: Precise control and customization of material properties. Enhanced compatibility with other materials through graft chemistry. Improved mechanical strength and chemical resistance. Effective for encapsulating poorly water-soluble compounds, such as drugs. Disadvantages: Complex synthesis process, requiring careful adjustment of monomer composition and sequence.
CS-g-SBMA grafted with SBMA	Drug loaded with antibacterial properties.	The synthesis and grafting of the superabsorbent chitosan-based hydrogel sponge were evaluated using ^1^H NMR and FTIR, respectively. X-ray diffraction (XRD) analysis indicated that the grafted copolymers are completely amorphous.	[47]	

mPEG: poly (ethylene glycol) methyl ether, PLLA: poly(L-lactide), PCL: poly(ε-caprolactone), βTCP: β-tricalcium phosphate, hCAp: carbonated hydroxyapatite with hexagonal crystallite shape, pCAp: carbonated hydroxyapatite with plate-like crystallite shape, PBMA-b-PMAA-b-PBMA: poly(butyl methacry-late)-b-poly(methacrylic acid)-b-poly-(butyl methacrylate), P(MF-alt-VBP): fatty acid attached maleimide and functionalized styrene, PCL-Fc: poly(ε-caprolactone) with ferrocene, POEGMA: poly(oligo ethylene glycol) methacrylates, pOEGMA-b-pLMA-Br: polar oligo (ethylene glycol)methacrylate and nonpolar lauryl methac-rylate from brominated dendritic macroinitiators, PBAx-co-PNMAy: poly{(n-butyl acrylate)-co-[N-(hydroxymethyl)-acrylamide]}, PDMAEMA: poly(dimethylamino ethyl methacrylate), PDIPAEMA: poly((2-(diisopropylamino) ethyl methacrylate), PCL-graft-pHPMA: poly(ε-caprolactone)-graft-(poly-N-(2-hydroxypropyl) methacrylamide), CS-g-SBMA: chitosan copolymers, SBMA: [2-(methacryloyloxy)ethyl]dimethyl-(3-sulfopropyl)ammonium hydroxide.

Triblock copolymer-based membranes in Pluronic polymersomes have also been evaluated for their ability to encapsulate compounds used in the design of in vitro enzymatic reactions. Confocal microscopy showed that these Pluronic-based polymersomes have a uniform size with an average diameter of 118 nm. Determination of fluorescent dye molecules and ion permeability indicated a molecular cut-off of approximately 500 Da and permeability towards K^+^ and Mg^2+^, which are essential for various enzymatic reactions [37]. In materials engineering, block ACs are employed to create nanocomposites with enhanced mechanical and thermal properties. For instance, block copolymers of polyester-b-PEPA were introduced into composite materials for bone surgery and orthopedic applications due to their improved strength and elasticity. Size exclusion chromatography (SEC) analysis of these polymers showed a uniform molecular weight distribution, while FTIR studies confirmed the chemical bonding of the ACs and the pCAp composite surface. SEM data demonstrated that ACs effectively dispersed pCAp particles during composite molding [38]. Another example is the use of poly(ethylene oxide)-poly(propylene oxide)-poly(ethylene oxide) block copolymer (PEO-PPO-PEO) to create tissue adhesives with thermosensitive properties, designed to reduce inflammation at human body temperature. The swelling ratio of the cured adhesives significantly decreased from 4 °C to 37 °C, and the inflammation ratios at 37 °C were relatively low, ranging from 8.4% to 22.5%. Additionally, the lap-shear adhesion strength on moist porcine skin ranged from 29.2 to 67.1 kPa, surpassing that of commercial fibrin glue, which ranges from 9 to 15 kPa [48]. In the field of nanotechnology, ACs can be used to create hybrid structures with unique properties by combining organic and inorganic characteristics for applications in catalysts, sensors, and optoelectronic devices [39]. For example, Kolhe et al. (2024) developed an AC of poly(ethylene oxide-block-methyl methacrylate) that was immobilized with antibodies in a recessed nano-disk array electrode, aimed at fabricating an electro-immunosensor for monitoring the overuse of antibiotics in food products derived from animals [49].

### 2.2. Alternating ACs

In the case of alternating ACs, the self-assembly behavior of methoxy poly(ethylene glycol)-functionalized styrene and fatty-acid-attached maleimide monomers was investigated. It was found that these ACs form micelle and vesicle nanostructures in water due to the amphipathic nature of the side chains and produce reverse micelles in hexane due to the presence of hydrophobic fatty acid monomers. ^1^H NMR findings suggest the development of a core–shell type nanostructure in aqueous solution, with a hydrophobic fatty acid core enveloped by a mPEG layer. The morphologies of the copolymers were spherical, with average sizes of 82 ± 18, 108 ± 22 and 48 ± 12 nm. The authors discuss the potential advantages of these synthesized copolymers for hydrophilic drug loading abilities [40]. Computational studies have also evaluated the self-assembly behavior of sequence-controlled ACs with alternating rod and coil side chains, particularly for drug loading by isolating two reactive drugs [50]. The study showed that alternating ACs self-assemble into onion-like vesicles through a rod-to-coil conformational transition of the rigid side chains. The regular alternation in these ACs can improve the compatibility and miscibility of components compared to other types of copolymers, such as random copolymers. Consequently, these copolymers act as emulsifiers and stabilizers. For example, [41] used styrene-maleic acid alternating ACs to solubilize and stabilize membrane proteins. These copolymers can solubilize lipid bilayers, forming nanodiscs that extract membrane proteins directly from cells in a water-soluble form, preserving them in a near-native environment suitable for further structural and functional studies. Interactions between lipids and ACs were investigated using FTIR spectroscopy and ^1^H NMR. It was discovered that the phenyl groups of the copolymers intercalate between the lipid acyl chains, oriented perpendicular to the lipid plane. Additionally, carboxyl groups engage in electrostatic interactions with the head groups of lipids located in the outer layer of the nanodisc. DLS analyses reported that the size of these nanodiscs is on the order of 10 nm.

### 2.3. Star Block ACs

Star block copolymer configuration offers high functionality, high chain density, and superior encapsulation ability compared to linear copolymers. These ACs can significantly enhance the physical, chemical, and mechanical properties of the material, such as viscosity, solubility, micelle-forming ability, and wear resistance [51,52]. One of the most studied applications of star ACs is in the biomedical field, including anticancer drug delivery, diagnostic therapy, and photodynamic therapy (PDT) [42,53,54]. For example, the application of polar oligo(ethylene glycol) methacrylate and nonpolar lauryl methacrylate-based star block copolymers, such as 2,2-bis(hydroxymethyl) propionic acid-star block copolymers (pOEGMA-Br and pOEGMA-b-pLMA-Br-star), has been explored in transdermal drug delivery. These ACs form reverse micelles and demonstrate potential for encapsulating fluorescein-isothiocyanate-labelled bovine serum albumin in nonpolar media, as well as their transdermal carrier capabilities using porcine skin. The number of repeat units for 1-arm, 6-arm, and 12-arm copolymers were determined by GPC and ^1^H NMR, with block ratios of 1.9, 1.5, and 1.2, respectively. DLS analyses revealed aggregate sizes between 175 and 250 nm for the 1-arm copolymer, which was supported by TEM analysis showing spherical structures of 700 and 800 nm. In contrast, TEM and DLS analyses of the 6-arm and 12-arm polymers showed smaller aggregates [43].

### 2.4. Statistical ACs

Statistical copolymers typically do not phase separate unless the components are very dissimilar in nature. This variability in monomer composition and distribution can result in a wide range of mechanical properties, from rigid to flexible [33,34,55,56,57]. Unusual copolymer assemblies, such as giant vesicles, vesicles, spheres, honeycomb films, and supramolecular hydrogels, have also been reported for the aggregation of amphiphilic statistical copolymers [58,59]. An interesting study recently reported the synthesis of self-crosslinkable poly{(n-butyl acrylate)-co-[N-(hydroxymethyl)-acrylamide]} (PBAx-co-PNMAy) ACs for developing self-healing materials. The mechanical properties and self-healing efficiency of these materials can be readily adjusted by controlling the monomer ratios and varying the self-cross-linking reaction conditions, making them adaptable for different applications. ^1^H NMR showed ratios in the copolymer systems between 8:2 and 7:3. SEC analysis indicated that the number average molecular weight ranged between 11,100 and 15,000, and FTIR confirmed the self-cross-linking behavior [44]. Statistical copolymers also have applications in the biomedical field. For instance, Makri and Pispas (2024) synthesized a statistical copolymer of poly(dimethylamino ethyl methacrylate) and poly((2-(diisopropylamino) ethyl methacrylate) [45]. The study investigated the behavior and properties of the resulting micelles and aggregates in relation to pH, temperature, and ionic strength of the aqueous solutions. The findings revealed intriguing behaviors and novel properties that make these structures valuable as nanocarriers for pharmaceutical compounds. SEC analysis demonstrated the successful completion of the polymerization process. Additionally, ^1^H NMR and FTIR experiments were performed to identify the chemical structure and composition of the copolymers, confirming the incorporation of both monomers into the copolymers.

### 2.5. Graft ACs

Graft ACs allow for precise control and tailoring of material properties by selecting and adjusting the monomer composition and sequence. Due to their versatility and tunability, these copolymers can enhance compatibility with other materials based on graft chemistry, as well as improve mechanical strength and chemical resistance [20]. For example, graft ACs composed of pegylated copoly(lactides) have been developed to exhibit reduced brittleness, increased hydrophilicity, and enhanced water absorption, making them suitable for use in implants. 

The branched structure and PEG to PLLA ratio in the copolymer units (30:70 or 50:50) were determined by ^1^H-NMR. The GPC data indicated that the average molecular weight of the copolylactides ranged from 8400 to 12,400. SEM analysis revealed a rough surface, likely due to microphase separation between the hydrophilic PEG segments and the hydrophobic lactide residues [60]. Another application of these ACs is in the encapsulation of poorly water-soluble compounds, such as drugs. Bláhová et al. (2020) demonstrated an effective dual delivery system using graft copolymers to concurrently encapsulate a hydrophobic drug, the Bcl-2 inhibitor ABT-199, and a pH-sensitive conjugate of another chemotherapeutic agent, doxorubicin (DOX) [46]. They confirmed the formation of micelles from PCL-graft-pHPMA copolymers using FFF, with nanoaggregate hydrodynamic diameters ranging from 23 to 32 nm. The extended stability of the graft copolymer and the high stability of its micelles were assessed using SEC, DLS, and ^1^H NMR, both in the presence and absence of lipase [46]. Chitosan copolymers grafted with [2-(methacryloyloxy)ethyl]dimethyl-(3-sulfopropyl) (SBMA) ammonium hydroxide were also evaluated as carriers for the antifungal drug caspofungin. These copolymers demonstrated significant potential for developing patches with antimicrobial and prolonged antifungal properties. The successful synthesis of these hydrogels and grafting of copolymers were verified by ^1^H NMR and FTIR, respectively. XRD analysis showed that grafted co-polymers are entirely amorphous [47].

### 2.6. AC Nanostructures 

Based on the studies discussed, it is evident that the aggregation of ACs into vesicles, micelles, and other nanoaggregates is crucial for their potential applications. Researchers have extensively focused on the synthesis, design, and characterization of these materials, examining parameters such as pH, temperature, polymer concentration, composition, and environmental conditions. Micelles, for example, consist of a hydrophobic core and a hydrophilic outer shell. They form when the concentration of the amphiphilic copolymer exceeds the CMC, the minimum concentration required for micelle formation. Factors influencing the formation of micelles include (i) hydrophobicity, (ii) glass transition temperature, (iii) degree of crystallinity, and (iv) the length ratio of hydrophilic to hydrophobic parts. Polymeric micelles are generally more stable than those formed by low-molecular-weight surfactants due to the interlocking of core-forming segments, which contribute to their thermodynamic and kinetic stability [20,21,61,62].

Conversely, vesicles are hollow spherical structures on the nanometer scale. They form when amphiphilic components, such as lipids or copolymers, aggregate in water. In this process, the hydrophilic regions orient outward, while the hydrophobic regions face inward, creating a lipid bilayer or a polymeric membrane that encapsulates an interior space [21,63]. Vesicles have the notable ability to encapsulate both hydrophilic and hydrophobic compounds, making them valuable for drug delivery and controlled release of bioactive substances [16,21,37,64]. Their stability is influenced by several factors, including (i) bilayer composition, (ii) vesicle size, (iii) component concentration, and (iv) environmental conditions [39,59,65]. For a comprehensive discussion on hydrogel classification and properties, see [66]. 

On the other hand, nanofibers are elongated polymeric structures formed through self-assembly processes, such as seed-driven crystallization, which enables control over their length and dispersion. Their structure imparts specific properties to the copolymers, including stability and high functionalization capacity, which enhance their load-bearing capabilities and responsiveness to external stimuli. The chemistry of the polymer segments, solvent conditions, and temperature can influence the morphology and properties of the resulting nanofibers, expanding their potential applications [67,68]. It is important to highlight that the self-assembly of ACs into various nanostructures is a crucial property for developing versatile carrier platforms for therapeutic delivery. These materials can form micelles that encapsulate hydrophobic drugs within their core, while vesicles can encapsulate both hydrophobic and hydrophilic drugs [17,46,62]. This encapsulation process not only enhances the solubility of poorly soluble drugs but also offers protection against degradation and enables controlled drug release, which is essential for optimizing therapeutic efficacy. Moreover, the ability to tailor the structure and composition of copolymers allows for selective adjustment of drug loading and release according to specific application needs [46,62,65]. Finally, the characterization of ACs and their nanoaggregates is critical for understanding their properties and behaviors, and the characteristics of AC NMs strongly influence their application in skin disease treatments.

## 3. ACs Applied Drug Delivery for Skin Cancer

The use of these NMs offers several advantages in skin treatments, including (i) protection and stabilization of active compounds against environmental conditions, preventing premature degradation; (ii) their nontoxic, biocompatible, and highly biodegradable nature; and (iii) enhanced cutaneous penetration and reduced systemic absorption, among others. ACs nanostructures are particularly effective for transporting water-insoluble drugs, enabling either passive or active drug targeting. Various methodologies have been developed for drug incorporation, which can be classified into two main categories: (i) encapsulation within the NMs by chemical interaction, and (ii) chemical conjugation of the drug. Table 2 provides examples of ACs used for transporting anticancer drugs. Among these methods, encapsulating drugs into the hydrophobic core of NMs is the most developed and straightforward approach, facilitating slow drug release. The main results and advancements in using ACs for cancer drug encapsulation and chemical conjugation are highlighted below.

### 3.1. ACs Applied in the Cancer Drug Encapsulation

**Figure 3:**
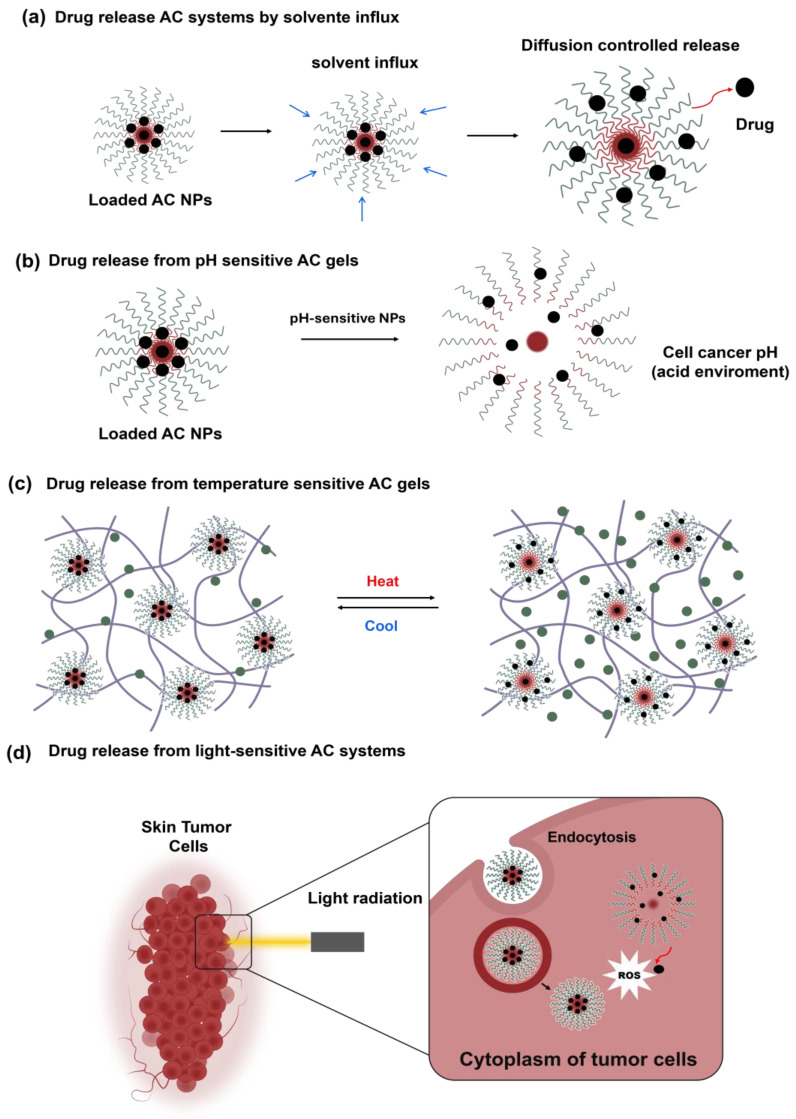
Illustration of different drug release mechanisms on AC NMs: (**a**) Controlled drug release by solvent influx. (**b**) Drug release triggered by pH changes. (**c**) Drug release in response to temperature variation. (**d**) Drug release from ACs systems combined with phototherapy to produce reactive oxygen species (ROS) in tumor cells.

Pluronic copolymers can form aggregates in aqueous solutions with hydrophobic cores, effectively encapsulating hydrophobic compounds like DOX, a drug used in cancer treatment via intravenous injection. Behera et al. (2021) investigated the interaction of Pluronic F127 and P123 aggregates with DOX using fluorescence spectroscopy and quenching experiments. Their findings revealed that the quenching constant was higher in F127 micelles compared to P123 micelles [69]. This difference is attributed to the greater hydrophilicity of F127 micelles, which leads to more compact aggregates that better facilitate DOX encapsulation. Additionally, incorporating the drug into Pluronic micelles increased the zeta potential, indicating that, besides hydrophobic interactions, electrostatic interactions between the micelles and DOX are also occurring. Pluronic has also been employed to form microemulsions (oil-in-water Pluronic 127) for DOX release. These microemulsions, with an approximate size of 7 nm as determined by DLS, release the drug sustainably over 24 h (~70%) at pH 7. Kinetic modeling of the in vitro release data suggests that the Korsmeyer–Peppas model best describes the release process. In the presence of water, the microemulsion swells, gradually expanding and allowing the drug to diffuse through the membrane (Figure 3a). Furthermore, the nanocarrier demonstrated an effective cytotoxic effect against C152 human oral squamous carcinoma cells, reducing cell viability from approximately 40% with DOX alone to less than 10% with the microemulsion after 48 h of treatment, thus exhibiting a potent cell-killing effect against carcinoma cells [70].

**Table 2:** In vitro drug releases and cytotoxicity results again skin cancer cells.

Formulation	Active Compound	Outcomes	Ref.
ACs applied in the cancer drug encapsulation			
(LPGE-ss) glutathione-responsive amphiphilic copolymers	DOX	LPGE micelles exhibited selective and enhanced antitumor activity compared to free DOX, reducing cell viability by approximately 50%, down to around 30%.	[71]
Nanogel of folic acid-Pluronic F-127	Triptolide	The nanogel formulation demonstrated significantly higher endocytosis capacity compared to the micelle formulation in human hepatocarcinoma (HepG2) cells.	[72]
Liposomes of Pluronic/pegylated lipids	ribonuclease	The liposomes exhibited nanometric dimensions (316 nm) and released 82% of the ribonuclease after 21 h. Their cytotoxicity against skin cells was higher than that of free ribonuclease, attributed to the enhanced endocytic cellular uptake of the particles.	[73]
PBEP-b-PBenEP nanoaggregates	PTX	The nanoaggregates loaded 4.3% of PTX and released 80% of the drug within 24 h. Additionally, they reduced the viability of HepG2 cells to approximately 25%.	[74]
PEG-b-PTMC-co-PDTC-b-PEI polymersomes	Cyclic dinucleotide-Cy3-labeled linear	The polymersomes enhanced tumor retention and controlled the release of cyclic dinucleotide (a Cy3-labeled linear form), inducing a stronger and longer-lasting immunotherapeutic effect.	[75]
Chitosan/Pluronic F-127 nanogel	Capecitabine	The nanogel exhibited an average size of 123 nm and released 40% of the encapsulated drug within 24 h. Additionally, it reduced the viability of HaCaT skin cells to 20% and demonstrated effective transdermal delivery in porcine tissue.	[76]
CA-PLGA-TPGS NPs	PTX	NPs were formed using the nanoprecipitation method, resulting in spherical materials with average hydrodynamic sizes ranging from 125.6 to 179.5 nm. The NPs exhibited high size stability for 90 days. Additionally, they released PTX in a controlled manner over 14 days (~45%) and demonstrated a cellular uptake efficiency of 50% in A875 melanoma cells.	[77]
PMPC_25_-PDPA_70_ polymersomes	DOX	Polymersomes were non-toxic to healthy cells and demonstrated a high release capacity, with less than 2% of encapsulated DOX released in 1400 s. The nanoformulation exhibited high cellular internalization in melanoma cells, leading to significant cell death within 24 h.	[78]
PEG-DCA micelles	DOX	The micelles were formed through a self-assembly process. They released 20% of the DOX over 70 h and reduced melanoma cell viability to less than 20%.	[79]
AC with chemically conjugated drug			
CPT-S-S-PEG-CUR	CUR/CPT	The amphiphilic dual-drug conjugated polymer (CPT-S-S-PEG-CUR) formed nanometric particles (~100 nm) in the presence of tannic acid, which acts as a crosslinker. The NM demonstrated effective cellular uptake in AsPC1 cancer cells, reducing cell viability to approximately 25%.	[80]
PVP NPs	DR5-B protein	PVP NPs exhibited greater cytotoxic effects compared to DR5-B protein in carcinoma HCT116 and HT29 cell lines, reducing cell viability to less than 10%.	[81]
mPEG-b-PAE-cis-DOX micelles	DOX	DOX is released from the micelles at acidic pH, with nearly 100% of the drug being released within 20 h. Additionally, the micelles reduce cell viability to less than 20%.	[82]

CPT: camptothecin, CUR: curcumine, PTX: paclitaxel, LPGE: linear polyglycidol, PAE: poly (β-amino esters), PBenEP: 2-(but-3-en-1-yloxy)-1,3,2-dioxaphospholane 2-oxide, PBEP: 2-butoxy-1,3,2-dioxaphospholane 2-oxide, PEI: polyethyleneimine; PEG: poly(ethylene glycol), mPEG: poly (ethylene glycol) methyl ether, PTMC: poly(dithiolane trimethylene carbonate, PDTC: trimethylene carbonate), PVP: poly(N-vinylpyrrolidone, PLGA: poly(lactide-co-glycolide), TPGS: tocopherylpolyethylene glicol, DCA: droxydodecanoic acid.

Pluronic can also be utilized to form nanogels, which are polymeric matrices crosslinked either chemically or physically, resulting in NMs sensitive to external conditions. Examples of crosslinkers used with Pluronic to create nanogels include heparin [83], chlorin e6 photosensitizer [84], and folic acid. For instance, Yin et al. (2019) employed folic acid as a physical crosslinker to form Pluronic 127 nanogels [72]. Physical crosslinkers facilitate noncovalent interactions such as hydrogen bonds, hydrophobic and van der Waals forces, and electrostatic interactions [85]. These nanogels exhibit sizes ranging from 15.7 to 68.6 nm and demonstrate a higher triptolide-loading rate (2.19%) compared to Pluronic nanomicelles. Endocytosis assays in HepG2 cells revealed that nanogels have a higher endocytosis rate than polymer micelles, leading to a 50% increase in drug accumulation in tumor cells.

Combining ACs with other compounds can significantly enhance drug loading capacity. For instance, surfactants such as dialkyl, alkyl, and cholesterol excipients have been used to form NMs with ACs. Faheela and Malathi (2022) developed niosomes—vesicular systems created with non-ionic amphiphilic surfactants having varying HLB values—using Pluronic P123 and cholesterol [86]. These niosomes, with an approximate size of 200 nm, demonstrated a high DOX entrapment efficiency of 88%. In vitro release studies at pH 7 revealed a slow release of DOX from the Pluronic P123/cholesterol niosomes, with approximately 80% of the drug being released over 100 h. Similarly, hybrid micelles composed of Pluronic 127 and P123 with phenyl boric acid were used for DOX delivery. In this system, DOX was grafted to P123 to control its release and make the system responsive to acidic environments. The in vitro release studies showed that DOX was released only under acidic conditions, with approximately 100% of the drug released over 100 h, indicating that the conjugated-acid-sensitive drug (CAD) was hydrolyzed, allowing free DOX to diffuse from the core aggregate [87]. Although these NMs have not been tested on skin cancer cells, they have exhibited cytotoxicity against MCF-7/ADR cells (breast cancer cells), suggesting potential for drug delivery and cancer cell apoptosis.

### 3.2. ACs Chemically Conjugate with the Drug

The previous examples illustrate the effectiveness of AC systems in releasing anticancer drugs for skin treatment, demonstrating significant cytotoxic effects. However, to enhance controlled release and increase the number of active compounds encapsulated, chemically conjugating ACs with the drug presents a compelling alternative. Despite this, there are limited methodologies for applying AC nanostructures, primarily due to the challenges associated with successful chemical modifications. For instance, Laskar et al. (2023) conjugated an amphiphilic polymer (CPT-S-S-PEG-CUR) using both an ester bond and a redox-sensitive disulfide (-S-S-) linkage with a PEG chain through two separate reactions [80]. The first reaction involved thiolate CPT, followed by the use of an amphiphilic dual-drug conjugated polymer with a PEG-based heterobifunctional crosslinker. Tannic acid was also utilized as a physical cross-linker to form stable NPs (~100 nm). These NPs demonstrated high cytotoxicity against AsPC1 and SW480 cancer cells, reducing cell viability by approximately 20% compared to the individual drugs CPT and CUR. The chemically conjugated drug–AC systems offer advantages such as enhanced stability in bloodstream circulation, increased biocompatibility, and reduced drug toxicity in the body. Furthermore, these NMs accumulate effectively at targeted sites rather than in normal tissues, facilitating efficient drug release and improved cancer cell killing [88,89]. 

DOX has also been conjugated to an amphiphilic diblock copolymer, poly(ethylene glycol) methyl ether-b-poly(β-amino esters), through an acid-labile cis-aconityl moiety (mPEG-b-PAE-cis-DOX) [82]. This drug-copolymer system exhibits self-assembly characteristics, forming polymeric micelles with a low CMC of 3.6 mg/L. The NMs effectively control the release of DOX at pH 7.4, delivering approximately 10% of the drug over 50 h. This release rate is lower compared to other DOX-encapsulating formulations, such as micelles of poly(β-amino ester)-graft-phosphorylcholine, which release 20% of DOX under the same conditions [90]. However, the mPEG-b-PAE-cis-DOX micelles are sensitive to acidic pH, which induces ionization of the tertiary amine residues in the PAE segment. This ionization leads to a shift from hydrophobic to hydrophilic properties, altering the micelle structure and enhancing the release rate of DOX from the micelle core. Micelles without DOX did not exhibit significant cytotoxicity against normal NIH 3T3 cells, indicating their nontoxic nature. In contrast, micelles with DOX reduced cell viability to approximately 10% in B16F10 melanoma cells. This was confirmed by confocal laser scanning microscopy (CLSM), which demonstrated that the micelles were primarily taken up by the cytoplasm. The chemical conjugation of DOX with the polymer improves therapeutic efficacy in B16F10-bearing mice, with both micelle-encapsulated DOX and DOX-loaded micelles reducing tumor weight to 0.1 g and 0.2 g, respectively, compared to 0.3 g for free DOX formulations. This demonstrates that the micelle systems are more effective than free DOX in treating tumors.

### 3.3. Controlled Release of Skin Cancer Drug by Stimuli-Responsive Materials

Recently, research has focused on developing AC NMs with controlled release mechanisms responsive to stimuli such as pH, temperature, and light (Figure 3). These systems can be combined with other cancer therapies, such as photothermal treatment, to enhance the effectiveness of apoptosis in cancer cells (Table 3). pH is a crucial factor for AC NMs because the extracellular environment of tumors is more acidic compared to normal tissue and blood (pH 7.4). Additionally, endosomes and lysosomes within cells also have acidic microenvironments (pH 5–5.5) [91,92]. Consequently, when ACs NMs enter cancer cells via endocytosis, they encounter this acidic environment, which induces conformational changes in the NMs. These changes facilitate drug release within the cancer cell, significantly enhancing the therapeutic effect [82,93,94]. 

To form AC NMs sensitive to pH, it is crucial to incorporate ionizable groups such as carboxyl, amino, sulfonate, and imidazolyl groups into the ACs [91,95]. Variations in pH, particularly at the low pH values typical of cancer cells, lead to the protonation of these functional groups. This protonation alters the hydrophilic–hydrophobic balance within the NMs, resulting in their disassembly and the subsequent release of active compounds. For instance, poly(propyl methacrylate-co-glucosamine/histidine/DOX) (P(PAA-co-GLU/HIS/DOX)), with a size of less than 100 nm, was employed for the controlled release of DOX [96]. Under acidic conditions, the amino groups of glucosamine become protonated, which alters the aggregate structure and results in approximately 60% drug release over 50 h. Another example involves biopolymers like chitosan, which is modified by grafting with poly(ethylene glycol) monomethacrylate (PEGMA) and hydrophobic deoxycholic acid. This modification creates amphiphilic polymers that form core-shell structures (~200 nm in diameter) with high drug concentration (PTX > 0.5 mg/mL). Under acidic conditions, the -NH₂ groups of chitosan become protonated, causing the polymer chains to expand due to repulsive forces and promoting PTX release (~12% over 4 h) [97]. Conversely, enzymes can facilitate drug release from ACs NMs by degrading the aggregates and releasing the active compounds. For example, hyaluronic acid (HA) conjugated with the hydrophobic moiety 5β-cholanic acid (5β-CA) self-assembled into HA-based NPs (HANPs) for controlled release of PTX against various skin cancer cell lines, including MDA-MB-435 melanoma, adenocarcinoma A549, and HepG2 cells [98].

**Table 3:** Recent application of the stimuli-responsive and synergic effect of AC NMs on skin cancer treatment.

Formulation	Active Compound	Outcomes	Ref.
Controlled release by stimuli-responsive			
Pluronic F127-chitosan nanogel	DOX	The NPs exhibited pH responsiveness, releasing DOX at acidic pH levels (5 and 6). Additionally, the nanogel demonstrated a better therapeutic effect compared to the free drug. The nanogel itself did not show toxic effects within the tested range (0.05–1 mg/mL) against HeLa cells.	[99]
P(Glu-b-NADA) NPs	THPP	The NPs exhibited sizes ranging from 180 to 200 nm and were responsive to acidic pH (5.5), releasing 100% of the drug within 24 h. In vitro cell assays demonstrated a high capacity to kill HeLa carcinoma cells, with an IC50 of less than 5%. The drug was detected in the epidermis and SC layers.	[100]
PNIPAM-b-PCL thermosensitive film	DOX	The pore size in the films was adjusted by varying the temperature between 10 °C and 40 °C, which enabled controlled release of DOX. The films demonstrated a loading capacity of 78% for DOX and achieved 80% cell inhibition in B16-F10 skin cancer cells.	[101]
PNIPAM–*b*-PEI microgel	DOX	The pH- and thermo-responsive microgel was effective against HepG2 cells. It released nearly 60% of its content at an acidic pH and 42 °C. Both pH and temperature changes altered the microgel’s structure, facilitating drug release.	[102]
Try-PLGA-PEG-PLGA-Try micelles	DOX	Try-PLGA-PEG-PLGA-Try micelles, featuring hydrophobic blocks (PLGA and Try) and a hydrophilic PEG corona, showed low cytotoxicity against NIH-3T3 cells, with 95.33% viability. When loaded with DOX, these micelles demonstrated significant anticancer efficacy, reducing cell viability by 58%. Both radiation and pH variations induced drug release from the micelles.	[103]
Synergic effect on cancer treatment			
Gold NPs/Pluronic F-127 nanogel	DOX	The nanogel demonstrated sustained release of AuNPs and DOX and effectively inhibited cell viability in mouse melanoma (B16) under radiation. Additionally, the nanogel exhibited safety in vivo, showing no adverse effects on mouse skin.	[104]
BDTO nanoagregates	Tariquidar	The nanoaggregates induced both PDT and chemotherapy, exhibiting a synergistic lethal effect on SKOV-3/MDR cells and enhancing tumor growth inhibition.	[105]
PNIPAM_65_-SS-PDOPA (PVN)	DOX and IR780 iodide	The PVN NPs were uniform (~100 nm) and, under laser irradiation, exhibited a synergistic effect of photothermal (IR780 iodide) and chemical (DOX) cytotoxicity against carcinoma CT26 cells, reducing cell viability to approximately 10%.	[106]
DOX-GNRs-PNIPAM@PEG-PLA micelles	DOX	Temperature and in situ near-infrared (NIR) light stimulation induced controlled DOX release and reduced cell viability to less than 10% in DOX-GNRs-PNIPAM@PEG-PLA micelles, which have an approximate size of 100 nm.	[94]

Glu: poly (L-glutamic acid), NADA: poly(Nγ-acetyl-L-2,4-diaminobutyric acid), THPP: 5,10,15,20-tetrakis(3-hydroxyphenyl) porphyrin, PNIPAM: poly(N-isopropyla-crylamide), PCL: polycaprolactone, PEI: polyethyleneimine, PLGA: poly(latic acid-co-glycolic acid), PEG: poly (ethylene glycol), Try: tryptophan, BDTO: 4,8-bis((2-ethylhexyl)oxy)benzo[1,2-b:4,5-b’]dithiophene-1,1,5,5-tetraoxide, PNIPAM65: poly(N-isopropylacrylamide)_65_, PDOPA: poly(3,4-dihydroxy-L-phe-nylalanine)n, GNRs: gold nanorods, PLA: poly(D,L-lactide).

These NPs are sensitive to the enzyme hyaluronidase, which degrades the HANP particles and releases approximately 75% of the PTX within 4 h. In contrast, without enzymatic activity, the NMs release about 15% of the drug in the same time frame, thereby controlling the release process. Additionally, the NMs loaded with PTX demonstrated a 6- to 75-fold increase in the IC50. In vivo assays in mice showed that these NMs inhibited skin cancer growth six times more effectively than control groups, resulting in a 100% survival rate. Such strategies highlight how modifying the chemical structure and environment of AC NMs can significantly enhance drug release.

Physical stimuli, such as temperature and light, have also been explored to induce drug release from AC NMs. Temperature changes can trigger sol–gel and gel–sol transitions in these NMs. Typically, at lower temperatures, AC NMs are dispersed in solution but transition into a gel when the temperature increases. This property allows the formulations to be injected subcutaneously or applied to the skin. As the temperature rises, the gels revert to a sol (suspension), altering intermolecular interactions and releasing active compounds in the tumor environment. An interesting system formulated by Cao et al. (2021) involved a smart transdermal formulation using a poly(D,L-lactide-co-glycolide)-b-poly(ethylene glycol)-b-poly(D,L-lactide-co-glycolide) thermogel for the controlled release of 5-aminolevulinic acid (ALA) [107].

The researchers adjusted the polymer blending ratio to achieve a sol–gel transition temperature (T_gel_) < room temperature (T_air_) < gel–sol (suspension) temperature (T_sol_ (suspension)) < body temperature (T_body_) [107]. This formulation exhibited asymmetric behavior when applied to the skin, becoming a gel on the exterior while a sol (suspension) remained in contact with the skin. As a result, the drug in the formulation is released more effectively into the skin. The gels enhanced the cumulative permeation of ALA in porcine skin and reduced cell viability by approximately 50% in B16-F10 cells. The research discussed demonstrates that AC NMs offer a robust alternative for drug delivery to skin cancer cells. Additionally, other cancer treatments have been explored with AC NMs, such as PDT, which enhances drug efficacy in cancer treatment. PDT involves using photosensitizer compounds to produce ROS upon exposure to light, leading to cell death. AC NMs exploit the enhanced permeability and retention (EPR) effect of cancer cells and the poor efficiency of lymphatic drainage to accumulate in tumor tissue, thereby increasing the release of photosensitizers into the cells [108,109]. Oudin et al. (2019) utilized ACs based on poly(2-methyl-2-oxazoline) (PMMA) to release pheophorbide, a known photosensitizer, into skin cancer cells [110]. Cytotoxicity and phototoxicity assays on HCT-116 tumor cells revealed that while the PMMA-based ACs with pheophorbide showed minimal cytotoxicity, they produced a significant phototoxic effect, reducing cell viability to 25%. Combining temperature and light stimulation with PDT has also been explored. For instance, Jiang et al. (2019) developed AC NMs using the block copolymer P(AAm-co-AN-co-TPP)-b-POEGMA (where AAm is acrylamide, AN is acrylonitrile, TPP is tetraphenylporphyrin, and POEGMA is poly(oligo-(ethylene glycol) methacrylate)) to enhance the thermal responsiveness of TPP, which exhibits higher photoactivity [111]. The temperature of these NMs was increased by irradiation with an 808 nm laser, raising it to above 50 °C. This temperature increase led to NM dissociation and release of active compounds. The NMs decreased cell viability by approximately 50% in A549 cells over 8 h and reduced tumor weight in mice by 90% compared to the positive control group.

Recent research is focusing on enhancing drug release in skin cancer treatments by combining various methodologies with PDT using metallic NPs. These NPs produce ROS when illuminated in the presence of a photosensitizer. Qi et al. (2020) developed a poly(2-(hexamethyleneimino)ethyl methacrylate)-b-poly(ethylene glycol) monomethyl ether methacrylate)-b-poly(diethylenetriaminepentaacetic acid methacrylate)-b-poly(1-vinyl imidazole)-b-poly(4-vinylphenylboronic acid) (PC7A-PEG-DTPA-VI-PBA) micelle system combined with CdSeTe quantum dots (QDs) [112]. This system was designed to release DOX in response to pH stimulation and enhance PDT, as QDs generate hydroxyl radicals (·OH) and increase intracellular ROS levels. The nanoformulation, with a diameter of approximately 200 nm, released 20% of DOX at pH 7.4 and 80% at pH 5.0 within 25 h. In B16F10 cells, cell viability was about 50% without light stimulation but decreased to 30% with the combined effects of DOX and phototherapy. This synergistic effect was further validated by in vivo assays in mice, where the relative tumor volume decreased from 30 to 15 (V/Vo). These results suggest that the synergy between drug release and PDT significantly enhances the effectiveness of cancer treatment, potentially reducing the required dosage of chemotherapy drugs and minimizing their adverse effects.

## 4. Recent Advances in the Delivery of Drugs and Active Substances for Skin Diseases Using Amphiphilic Copolymers

Skin diseases encompass a wide range of conditions that affect the integrity and function of the largest organ in the human body. Among the most common skin diseases are acne, atopic dermatitis, psoriasis, and fungal infections [113]. Despite their varying severity and symptoms, these conditions share certain inflammatory processes and cellular alterations with skin cancer, one of the most aggressive and deadly forms of skin disease. The similarity between skin diseases and skin cancer lies in the deregulation of normal cellular functions, which can lead to uncontrolled cell proliferation. Both types of conditions can be influenced by genetic, environmental, and lifestyle factors, such as excessive exposure to ultraviolet radiation [114]. Continued research into the delivery of drugs and active substances, particularly through the use of ACs, offers new hope and solutions for the effective treatment of these dermatological conditions [115]. Table 4 provides a comprehensive and detailed overview of the versatility and potential of ACs in treating various skin diseases, summarizing how these innovative copolymers are revolutionizing skin condition treatments. It also highlights their role as promising vehicles for drug delivery, showcasing recent advances and their effectiveness in enhancing drug penetration and therapeutic action. The subsequent section reports on the application of ACs in treating different skin diseases.

### 4.1. Acne

Acne is a common skin condition characterized by the appearance of pimples, blackheads, and cysts, resulting from the obstruction of hair follicles and sebaceous glands. This blockage causes inflammation and, in severe cases, can lead to permanent scarring [116]. ACs offer a promising solution for acne treatment due to their unique ability to form micelles and vesicles that encapsulate active medications. This encapsulation allows for controlled and targeted release, improving penetration into the deeper layers of the skin [117]. These copolymers enhance the solubility and stability of therapeutic agents, facilitating the delivery of anti-inflammatory, antibacterial, and sebum-regulating medications directly to affected areas. By doing so, they reduce inflammation, combat bacterial infections, and normalize sebaceous gland activity, promoting more effective and faster healing of acne [118]. Significant advances have been made in acne treatment through drug delivery via ACs. For instance, mixed polymeric micelles developed with Pluronics F-68 and F-127, loaded with dapsone (DAP), have shown reduced skin irritation and minimized adverse systemic effects, thereby improving the safety and efficacy of DAP treatment. Optimized mixed micelles have also been explored for the topical delivery of DAP in anti-acne therapies, with studies demonstrating a significant improvement in DAP solubility, reaching up to 18.42 µg/mL and achieving 98.11% permeability within 6 h. Additionally, Carbopol gels used as gelling agents have exhibited thixotropic properties and low syneresis, with no signs of dermal toxicity in animal models [119]. These findings underline the potential of mixed micelles as effective and safe carriers for the topical delivery of DAP, highlighting their ability to improve therapeutic outcomes in acne treatment. In parallel, poly(ε-caprolactone)-poly(ethylene glycol)-poly(ε-caprolactone) (PCL-PEG-PCL) micelles loaded with lauric acid (LA) notably enhanced bactericidal activity against Propionibacterium acnes. The minimum inhibitory concentrations (MIC) and bactericidal concentrations (MBC) of LA in micelles decreased to 10 and 40 µg/mL, respectively, compared to 20 and 80 µg/mL for free LA, demonstrating greater therapeutic efficacy [120].

**Table 4:** AC application to drug release to skin disease (in vivo and in vitro results).

Amphiphilic Copolymer	Drug	Disease	Outcomes	Ref.
PLA-b-PEG	Cholecalciferol	Psoriasis	PLA-b-PEG NPs (0.28 µg/cm^2^) showed higher skin absorption compared to the formulation with lower PEG content (0.18 µg/cm^2^).	[121]
P(AlHEMA-co-MPEGMA)	VitC, ARB and 4nBRE	Skin hyperpigmentation	The heterografted copolymers exhibited “click” reaction efficiencies ranging from 17% to 70% or 32% to 50%, forming micelles that encapsulated VitC, ARB, and 4nBRE with loading efficiencies of 5% to 55%, 39% to 91%, and 42% to 98%, respectively. In vitro tests indicated a maximum release of these active ingredients within 10 to 240 min.	[122]
PMCC Nps	Glab	Melasma	NPs showed a significant reduction in IL-1α production without affecting cell viability. NPs containing 0.05% 1,2-decanediol (DD) sterilized nearly all methicillin-resistant *Staphylococcus aureus* within 120 min and Propionibacterium acnes within 30 min. Additionally, the NPs increased Glab concentration in the epidermis by more than threefold compared to conventional O/W micelles.	[123]
MPEG-PCL	CUR	Dermatitis	Micelles loaded with CUR exhibited a loading capacity of 12.14% and an encapsulation efficiency of 93.57%, increasing CUR’s solubility to 1.87 mg/mL—1.87 × 10^6^ times higher than native CUR. Supramolecular hydrogels (CUR-H) demonstrated continuous dissolution over 4.5 h and improved cutaneous deposition of CUR. In vivo studies revealed that CUR-H was more effective than dexamethasone ointments in reducing croton oil-induced ear edema.	[124]
MPEG-PCL	Hydrocortisone (HC)	Atopic dermatitis (AD)	A loading efficiency of 9.95 ± 0.12% and an encapsulation efficiency of 97.11 ± 0.96% were achieved. The micelles exhibited a monodisperse size distribution with a mean particle diameter of 25.3 ± 0.6 nm and a significantly higher permeation rate (1.47 μg/(cm^2^ h)) compared to the HC cream (0.16 μg/(cm^2^ h)). The cumulative permeation of HC demonstrated a 9.2-fold increase compared to the standard HC cream.	[125]
PEO-PPO	DAP	Acne	The mixed micelles demonstrated improved solubility for DAP, reaching up to 18.42 µg/mL, with a drug content exceeding 85%. Ex vivo permeation studies revealed that 98.11% of DAP was permeated from the micellar solution within 6 h, with a release rate of 16.57 µg/cm^2^/h. In comparison, simple gels showed a release rate of 9.94 µg/cm^2^/h and a cumulative release of 81.67% at 6 h.	[119]
Pluronic F127	TMN	Hypertrophic Scars	The nanomicelles released over 80% of the TMN within the first 30 min in vitro. High doses of TMN (100–150 μg) resulted in reduced epidermal thickness and improved collagen organization. Additionally, there was a decrease in the expression of key fibrosis markers, including α-SMA, COL1, and TGF-β1.	[126]
F127-(CS-LNA)_2_	AMB	Cutaneous mycosis	Characterization revealed interactions between AMB and the copolymer, with AMB present in an amorphous form within the micelles. The micelles demonstrated antifungal activity comparable to that of a commercial injection, but with a slower and more sustained release profile, releasing 44% of the drug after 48 h.	[127]

PLA: poly(lactic acid), PEG: poly(ethylene glycol), AlHEMA: poly(2-hydroxyethyl methacrylate, MPEGMA: methoxypoly(ethylene glycol)methacrylate), PMCC: poly(methacrylate methyl-co-n-butyl acrylate), MPEG: methoxy poly(ethylene glycol), PCL: poly(ε-caprolactone), PEO: poly(ethylene oxide), PPO: poly(propylene oxide), Pluronic F127: (poly(ethylene oxide)-poly(propylene oxide)-polyvinylpyrrolidone-polyvinyl alcohol), F127-(CS-LNA)_2_: micelles F127 copolymers with LNA (linoleic acid), CS: cetyltrimethylammonium chloride, Cholecalciferol: vitamin D3, VitC: Vitamin C, ARB: Arbutin, 4nBRE: 4-n-butylresorcinol, Glab: Glabridin, TMN: Tranilast, AMB: Amphotericin B.

Furthermore, electrostatic optimization of adapalene emulsions using copolymers with different contents of pendant amine groups resulted in significantly more stable emulsions, with a two- to three-fold stronger therapeutic effect against acne vulgaris in animal studies compared to emulsions prepared with anionic emulsifiers or without any emulsifier. This emulsion also notably inhibited macrophage expression, further highlighting its therapeutic potential [128].

### 4.2. Hyperpigmentation and Melasma

Hyperpigmentation and melasma are skin conditions characterized by darker areas and uneven skin tone. Hyperpigmentation can result from factors such as sun exposure, aging, skin trauma, or underlying medical conditions. Melasma, in particular, is marked by brown or gray spots that primarily appear on sun-exposed areas like the face and may be associated with hormonal changes, such as those occurring during pregnancy or with contraceptive use [129]. ACs have shown potential in treating hyperpigmentation and melasma due to their ability to enhance skin penetration and stabilize depigmenting agents. These copolymers can encapsulate active ingredients such as hydroquinone, kojic acid, or ascorbic acid, which are known for their properties in inhibiting melanin synthesis or promoting its removal [130]. When applied topically, ACs facilitate the controlled release of these ingredients into the deeper layers of the skin, where they work to reduce pigment production and improve the appearance of dark spots. In addition to their role in delivering depigmenting agents, ACs help maintain the stability of these active ingredients, protecting them from degradation and optimizing their effectiveness over time [131]. Heterograft copolymers have demonstrated loading efficiencies of up to 98% for active compounds such as VitC, ARB, and 4nBRE, with maximum release occurring within 10 to 240 min. These copolymers exhibited a “click” reaction efficiency ranging from 17% to 70%, or 32% to 50%, depending on the molecular weight of the polycaprolactone (PCL), forming micelles that encapsulate these active ingredients [122]. This approach could be further developed to create more effective and sustainable topical treatments for hyperpigmentation conditions, ensuring controlled release and greater treatment efficacy. Polymer systems based on amphiphilic graft copolymers, such as those designed to encapsulate ARB and VitC, demonstrate high encapsulation efficiency and provide rapid and controlled release of active agents in aqueous solutions, which is advantageous for cosmetic applications [132]. These formulations are ideal for products like masks, creams, and wraps as they enable the rapid release of antioxidants and skin-lightening agents, thus enhancing their cosmetic effectiveness.

Another significant advancement is the formulation of polymeric micelles formed by partially myristoylated carboxymethyl chitosan in combination with DD and Glab. This formulation showed a marked improvement in antimicrobial activity and inhibition of melanogenesis. For example, myristoylated carboxymethyl chitosan with 0.05% of DD (an antimicrobial agent) significantly reduced IL-1α production without affecting cell viability and eradicated nearly all methicillin-resistant *Staphylococcus aureus* (MRSA) bacteria within 120 min and Propionibacterium acnes within 30 min. Furthermore, the Glab-loaded micelles increased the concentration of Glab in the epidermis by more than three times compared to conventional emulsified micelles, enhancing skin brightness and indicating a greater inhibition of melanogenesis [123]. These findings underscore the versatility of these copolymers in dermatological applications.

### 4.3. Dermatitis

Dermatitis encompasses a diverse group of inflammatory skin conditions that can manifest as redness; itching; peeling; and, in severe cases, blisters and scabs. The most common types include atopic, contact, seborrheic, and nummular dermatitis [133]. These conditions can be triggered by allergens, chemical irritants, microorganisms, or genetic factors, often involving an exaggerated immune response. ACs have shown promise in the treatment of dermatitis due to their ability to encapsulate and release anti-inflammatory agents and emollients directly onto the affected area. When applied topically, these copolymers facilitate the penetration of active ingredients that can reduce inflammation, restore the skin barrier, and relieve symptoms such as itching and redness [134]. In the context of AD, recent research highlights the potential of advanced copolymer formulations for enhancing treatment efficacy. For instance, a study demonstrated the effectiveness of a HC-loaded micellar compound combined with a carbomer hydrogel. This formulation achieved an encapsulation efficiency of 97.11 ± 0.96% and a monodisperse distribution with a mean particle diameter of 25.3 ± 0.6 nm. It significantly increased the skin permeation rate to 1.47 μg/(cm^2^ h) and the accumulated amount of HC, compared to traditional HC cream, which had a permeation rate of only 0.16 μg/(cm^2^ h) [125]. Similarly, CUR, known for its anti-inflammatory properties, faces challenges due to its low water solubility and skin permeability. However, the use of methoxy poly(ethylene glycol)-block-poly(ε-caprolactone) (MPEG-PCL) to create CUR-loaded micelles has markedly improved its solubility and skin deposition. CUR-loaded supramolecular hydrogels (CUR-H) increased drug solubility to 1.87 × 10^6^ times that of native CUR and enhanced its anti-inflammatory efficacy in vivo. This formulation demonstrated continuous dissolution over 4.5 h and superior skin deposition compared to dexamethasone ointments. These findings underscore the potential of MPEG-PCL and α-cyclodextrin (α-CD) micelles as effective formulations for treating inflammatory skin conditions, offering improved drug delivery and therapeutic outcomes [124].

### 4.4. Psoriasis

ACs offer promising advancements in the treatment of psoriasis, enhancing topical therapies and potentially reducing the need for more aggressive oral treatments [135]. Psoriasis is a chronic condition marked by rapid skin cell proliferation, leading to the development of scaly, red plaques that cause itching and discomfort. It can be triggered by genetic and environmental factors and often affects areas such as elbows, knees, the scalp, and the lower back [136]. The use of ACs in encapsulating anti-inflammatory agents, such as corticosteroids or vitamin D analogs, has shown significant benefits in treating psoriasis. These copolymers facilitate targeted delivery of the medications, allowing for more effective reduction of inflammation and slowing of excessive cell proliferation. Additionally, ACs can help restore the skin barrier, thereby alleviating symptoms and improving the appearance of psoriatic lesions [137]. Recent research into NPs systems for psoriasis treatment has shown that adjusting the PEG content can significantly impact the cutaneous absorption of cholecalciferol. Higher PEG content in NPs, such as PLA-b-PEG5K and PLA-b-PEG10K, achieved better absorption rates (0.28 and 0.30 µg/cm^2^, respectively) compared to those with lower PEG content (PLA-b-PEG1K and PLA-b-PEG2K). This underscores the importance of optimizing NP composition based on skin conditions to enhance therapeutic efficacy [121]. Additionally, poly(ε-caprolactone)-poly(ethylene glycol)-poly(ε-caprolactone) micelles loaded with LA have been explored to improve the bioavailability and effectiveness of fluocinolone acetonide for psoriasis. These micelles demonstrated controlled drug release, effectively reducing psoriasis symptoms while maintaining anti-inflammatory activity. This approach highlights the potential of optimized micelles in achieving better therapeutic outcomes [138]. Another innovative approach involved developing telodendrimer-based nanocarriers for encapsulating methotrexate (MTX). This system achieved an MTX loading capacity of over 20% (*w*/*w*) in 20–30 nm particles, allowing for controlled drug release and excellent hemocompatibility. In animal models of psoriasis-like inflammation, MTX nanoformulations showed superior and long-lasting efficacy in reducing inflammation compared to free MTX, demonstrating the potential for these nanocarriers in improving psoriasis treatment [139].

### 4.5. Fungal Infections

Fungal infections of the skin, such as cutaneous mycosis caused by dermatophytes, yeasts like *Candida albicans*, and molds, can lead to symptoms such as redness, peeling, itching, and changes in skin texture. These infections can be challenging to treat due to the fungi’s ability to persist and proliferate in the skin [140]. ACs have been used to encapsulate antifungals, such as clotrimazole or terbinafine, to target areas where fungi usually proliferate, thereby reducing degradation and increasing the persistence of the antifungal treatment [141]. For example, micelles formed with F127 copolymers and linoleic acid (F127-(CS-LNA)_2_) have been used to encapsulate AMB. This formulation demonstrated high encapsulation efficiency (97.26 ± 2.85%) and loading capacity (12.51 ± 0.39%), with a controlled release of 44% of AMB over 48 h. Notably, this micellar formulation showed a 310-fold increase in cutaneous drug deposition compared to commercial liposomal gels and exhibited superior antifungal activity relative to existing commercial formulations [127]. These advances illustrate the potential of AC in improving the delivery and effectiveness of antifungal treatments, enhancing their ability to reach and treat fungal infections in the skin effectively. Furthermore, the luliconazole (LUL)-loaded polymeric micellar (P123/F127) hydrogel formulation (LUL-PM-CHG) exhibited a particle size of 226 nm and a micellar incorporation efficiency (MIE) of 88.38%. This system provided sustained release of LUL with prominent antifungal activity against Candida albicans, showing MIC values of 3.25 ng/mL and high inhibition of ROS production, indicating significant improvements in treatment effectiveness [142]. Finally, the Pluronic (P407)-loaded AMB thermoreversible gel (AmB-gel), developed for the treatment of dermal and vaginal candidiasis, demonstrated high porosity and controlled drug release with significant retention in the skin (960.0 µg/g/cm²) and vaginal mucosa (737.3 µg/g/cm²). This gel exhibited superior antifungal efficacy compared to free AmB against *Candida* spp. strains, as supported by AFM studies and showed excellent dermal tolerance without inducing irritation or altering biophysical properties [143].

### 4.6. Hypertrophic Scars (HS) and Keloids

HS and keloids are abnormal forms of skin scarring characterized by excessive growth of scar tissue after an injury. HS are raised and usually confined to the area of the original lesion, while keloids extend beyond the initial site and may be thicker and more prominent. These conditions can be triggered by burns, cuts, surgeries, severe acne, or even piercings and tattoos [144]. ACs can encapsulate active ingredients such as corticosteroids or agents that modulate cell proliferation. By doing so, they reduce inflammation, inhibit collagen overproduction, and promote proper remodeling of scar tissue, which can significantly improve the appearance and texture of scars [145]. Amphiphilic block copolymers of poly(L-glutamic acid)-b-poly(Nγ-acetyl-L-2,4-diaminobutyric acid) have also shown promising capacity for dermal delivery of photosensitizers such as meta-tetra(hydroxyphenyl)porphyrin (m-THPP). This copolymer not only improved drug penetration into the skin in ex vivo studies but also demonstrated controlled release and low cytotoxicity in HaCaT and HeLa cells, underscoring its potential as a topical delivery system [100]. The development of new technologies for cutaneous drug delivery has seen significant advances, especially with the use of AC. A recent study evaluated the efficacy of tranilast-loaded nanomicelles (TMN) in treating HS. The nanomicelles released more than 80% of the tranilast within the first 30 min in vitro. Higher doses of tranilast (100–150 μg) led to a reduction in epidermal thickness and improved collagen organization, accompanied by a decrease in the expression of proteins such as α-SMA, COL1, and TGF-β1, which are key markers of fibrosis [126]. These findings highlight the efficacy of TMN nanomicelles as a simple and cosmetically favorable solution for treating HS, offering effective protection and reliability in pathological scar therapy [126].

### 4.7. Bacteria Skin Diseases

Skin diseases caused by bacteria are common and encompass a variety of dermatological conditions. Among the most prevalent are cellulitis, impetigo, and infections caused by *Staphylococcus* and *Streptococcus* [146]. Frequently involving microorganisms such as *Staphylococcus aureus* and *Streptococcus pyogenes*, these bacteria can colonize intact skin or enter through cuts, abrasions, or compromised areas, triggering inflammatory responses that affect skin integrity and the patient’s well-being. Symptoms can include redness; inflammation; pain; and, in some cases, pus discharge [147]. ACs have emerged as effective tools for facilitating the delivery of antibacterial drugs into the deep layers of the skin. By doing so, they can help reduce bacterial load, alleviate symptoms, and promote faster recovery from bacterial skin infections [130]. Current research on drug delivery systems for skin diseases has explored innovative approaches using ACs. For example, an advanced antimicrobial system has been developed using PELI@BPQD-SNO NPs, where amphiphilic polymers derived from poly-L-lysine (PLL) and black phosphorus QDs (BPQDs) sensitized in the near-infrared (NIR) are combined with S-nitrosocysteamine (SNO) for the controlled release of nitric oxide (NO) [148]. This system demonstrated exceptional synergistic bactericidal efficacy, achieving a clearance rate of 99.6% in *Staphylococcus aureus*-infected subcutaneous abscess models, with remarkable recovery of infected wounds and efficient reduction of inflammation. Comb-shaped cationic polyethylene glycol (PEG) block polycarbonates have also been investigated. These polymers self-assemble into positively charged NPs of approximately 60 nm in size. The NPs exhibited notable antibacterial activity against Gram-negative, Gram-positive, and drug-resistant strains at low concentrations (MIC 64–128 μg/mL) and with low hemolysis (HC50 > 2000 μg/mL). In in vivo tests, the NPs significantly inhibited the growth of vancomycin-resistant bacteria when applied to wounds [149]. The innovative design of these comb-shaped amphiphilic polycarbonates offers a new strategy for developing effective antibacterial agents, highlighting their potential for the prevention and treatment of skin infections through delivery systems based on ACs [149].

### 4.8. Skin Autoimmune Disease

Vitiligo is a skin disease characterized by the loss of pigment in specific areas, resulting in white spots on various parts of the body. This condition occurs when melanocytes, the cells responsible for producing melanin, are destroyed or cease to function [150]. Although the exact cause of vitiligo is not fully understood, autoimmune, genetic, and environmental factors are thought to play significant roles [151]. ACs offer new hope for the treatment of vitiligo due to their ability to improve the delivery of therapeutic agents directly to affected melanocytes. These copolymers can encapsulate bioactive molecules that promote the regeneration of melanocytes or modulate the immune response to reduce the destruction of these cells, thereby improving repigmentation and color stability [152]. A notable example is the use of nanodispersions for the delivery of siRNA targeting tyrosinase (TyRP-1) in the treatment of vitiligo. This approach demonstrated a high rate of cellular uptake and a significant reduction of more than 80% in TyRP-1 protein levels, which is involved in the autoimmune destruction of melanocytes, thus showing great promise for topical therapy of vitiligo [153]. On the other hand, the application of catalase-loaded PEO_5_PPO_68_PEO_5_ polymersomes has proven effective in photoprotection and the treatment of skin disorders related to oxidative stress, such as skin aging and vitiligo. In this study, the optimized polymersomes exhibited a hydrodynamic diameter between 200 and 400 nm, an encapsulation efficiency of up to 4.72 ± 0.07%, and a polydispersity index ranging from 0.1 to 0.3, indicating significant potential for the topical delivery of catalase [154].

Cutaneous lupus erythematosus (CLE) is a form of lupus that affects the skin, causing lesions, rashes, and other changes, particularly in sun-exposed areas. This autoimmune disease occurs when the immune system mistakenly attacks healthy tissues, including the skin, resulting in inflammation and cellular damage [155]. Autoimmune skin diseases like CLE can be challenging to treat due to the need to suppress the immune response without compromising the patient’s overall health [156]. ACs offer an innovative solution in this context due to their ability to encapsulate medications such as corticosteroids, calcineurin inhibitors, or biological agents, providing controlled and sustained drug release. By efficiently penetrating the skin layers, these systems can reduce local inflammation and modulate the immune response, thereby minimizing systemic side effects [157]. Another autoimmune skin disease that can benefit from ACs is scleroderma, which is characterized by the hardening and thickening of the skin and can also affect internal organs [158]. ACs can be used to deliver drugs that improve skin elasticity and reduce fibrosis [159]. Similarly, pemphigus, a condition causing blisters and erosions on the skin and mucous membranes due to an autoimmune response against the proteins that hold skin cells together, can be effectively treated with ACs that facilitate the delivery of biological therapies and other immunosuppressive medications to control symptoms and prevent flares [160]. Another skin disease, dermatomyositis, which presents with muscle weakness and skin rashes, can also benefit from the use of ACs [161]. The administration of immunomodulators and anti-inflammatory agents through these copolymers can help control both the cutaneous and systemic manifestations of the disease [162].

ACs have emerged as promising tools to enhance therapeutic efficacy and reduce toxicity in the treatment of skin diseases. One study explored the use of poly(ethylene glycol)-b-poly(propylene sulfide) (PEG-b-PPS) phyllomicelles for the delivery of chloroquine (CQ) in systemic lupus erythematosus. These phyllomicelles were shown to improve drug specificity toward plasmacytoid dendritic cells, reducing type I interferon production and mitigating accumulation in the retinal pigment epithelium, a common adverse effect of soluble CQ [163]. In another approach, poly(lactic-co-glycolic acid) (PLGA) microspheres coated with PEG were developed and loaded with extracellular matrix (ECM)-degrading enzymes, such as collagenase and hyaluronidase. These microspheres exhibited a controlled release of enzymes for 10 days, reducing dermal thickness in scleroderma models without altering the mechanical properties of the skin [164]. Additionally, a recent study introduced a gene therapy gel that utilizes polyplex NPs in granular hydrogels for gene drug delivery. This innovative approach allows for regulated drug release, reducing toxicity and improving the efficiency of in vitro transfection. The gel also provides ease of storage, dosing, and administration of the treatment [165]. Finally, PLGA microspheres loaded with MTX have been designed for controlled and prolonged release in the treatment of dermal fibrosis. These microspheres demonstrate high encapsulation efficiency and a gradual release of MTX, which reduces type I collagen production and promotes matrix metalloproteinase-1 expression in dermal fibroblasts in vitro. Additionally, they show a significant reduction in fibrosis in vivo in mouse models [166]. Together, these advances highlight the versatility and effectiveness of ACs in drug delivery for skin diseases. From specific drug targeting to controlled enzyme release and gene therapies, these approaches represent significant steps toward more effective and less invasive treatments for various dermatological conditions [15].

Skin diseases encompass a wide variety of conditions, including eczema, hives, warts, and injuries from sun exposure, among others. These conditions can cause significant discomfort and affect the quality of life of those who suffer from them [167]. ACs can encapsulate therapeutic ingredients, which may reduce inflammation, relieve pruritus, and promote healing of damaged skin [114]. For instance, MTX-loaded micelles prepared with an amphiphilic cationic material, such as N,N-dimethyl-(N′,N′-di-stearoyl-1-ethyl)1,3-diaminopropane (DMSAP), have shown significant improvements in skin permeability and MTX deposition in the epidermis and upper dermis [168]. This approach not only enhances percutaneous delivery of MTX but also reduces associated side effects, offering a promising delivery system for the treatment of inflammatory and autoimmune skin diseases.

## 5. Conclusions and Prospects

ACs NMs are versatile and effective tools for drug delivery and active substance applications in the treatment of various dermatological conditions. Their ability to self-assemble into stable nanostructures, such as micelles, vesicles, and nanocapsules, results from their unique chemical structure, which combines hydrophobic and hydrophilic segments. This property facilitates the formation of diverse morphologies tailored to specific applications, optimizing drug solubility, protection, and controlled release. The inherent advantages of ACs, including their ability to protect and stabilize active compounds, their biocompatibility and biodegradability, and their effectiveness in enhancing skin penetration while minimizing systemic absorption, are crucial for developing advanced therapeutic platforms.

Drug incorporation methodologies, such as encapsulation and chemical conjugation, are employed to achieve controlled and sustained drug release, thereby optimizing therapeutic efficacy and reducing potential side effects. Advances in characterization techniques, including NMR, GPC, and electron microscopy, have been essential for understanding and improving the properties of these copolymers and their nanoaggregates. These tools enable detailed analysis of ACs’ structure and behavior, facilitating their design and application across various fields. Specifically, nanogels, niosomes, and hybrid micelles have shown high efficiency in drug loading and release, though further research is needed to assess their efficacy in treating skin cancer cells. Additionally, the ability of ACs to respond to external stimuli, such as changes in pH, temperature, and light, presents new opportunities for controlled drug release in tumor environments. Combining ACs with PDT has shown promising results, significantly enhancing treatment effectiveness against cancer by reducing cellular viability and tumor weight in animal models. Integrating these methodologies with metal NPs has also demonstrated potential for improving clinical outcomes by enhancing the generation of ROS and reducing the need for high doses of chemotherapy.

In the realm of dermatological diseases, ACs have demonstrated significant advancements in treatment efficacy for conditions such as acne, hyperpigmentation, dermatitis, psoriasis, and fungal infections. ACs improve drug solubility, stability, and penetration, enabling controlled and targeted release. This capability to encapsulate and efficiently deliver anti-inflammatory, depigmenting, antifungal, and antibiotic agents offers innovative and less invasive approaches for managing various skin conditions.

In conclusion, ACs represent a promising tool for enhancing the effectiveness and precision of dermatological treatments. Ongoing research and development in the synthesis, characterization, and application of these copolymers hold the potential to transform the treatment of various skin diseases, providing more effective and less invasive solutions for patients. The evolution of technology and understanding of ACs will undoubtedly expand their applications and improve clinical outcomes in the near future.
